# Myopic Strain: a normalized metric concept for assessing axial myopia

**DOI:** 10.3389/fopht.2025.1648686

**Published:** 2025-08-04

**Authors:** Qi Ren, Zhe Chu

**Affiliations:** ^1^ Department of Ophthalmology, The First Affiliated Hospital, Sun Yat-sen University, Guangzhou, China; ^2^ Shandong Provincial Key Laboratory of Ophthalmology, Qingdao, China

**Keywords:** axial myopia, Myopic Strain, retinal defocus, stress-strain index, spherical equivalent refractive error

## Abstract

Axial myopia is characterized by excessive axial elongation, traditionally quantified by axial length (AL). However, AL conflates the eye’s focal distance (adaptive to refractive power) with defocus distance (excessive axial elongation). In this study, we developed Myopic Strain, defined as the ratio of retinal defocus distance (ΔAL) to the eye’s focal length, yielding a normalized metric for assessing axial myopia. In an analysis of 242 eyes, ΔAL and Myopic Strain were derived from Morgan’s optometric model. Subsequently, the correlation between Myopic Strain and optical and biomechanical markers of myopia was analyzed. Finally, we analyzed the distinctive characteristics exhibited by Myopic Strain and the ratio of AL to corneal curvature radius (AL/CR) as AL increased. Results demonstrated that Myopic Strain showed significant correlations with optical and biomechanical markers of myopia—spherical equivalent refractive error (SER; *r* = –0.81) and stress-strain index (SSI; *r* = –0.30) (both *p* < 0.001). Correspondingly, Myopic Strain provided superior explanatory power for SER (*R²* = 0.65) and comparable power for SSI (*R²* = 0.09) (both *p* < 0.001). Furthermore, our analysis revealed a strong positive correlation between Myopic Strain and AL (*r* = 0.82, *p* < 0.001), concomitantly with a moderate positive correlation between AL/CR and AL (*r* = 0.64, *p* < 0.001). Notably, the theoretical emmetropization baseline of AL/CR exhibited an inverse relationship with AL. In conclusion, Myopic Strain emerges as a suitable normalized metric for assessing axial myopia severity.

## 1 Introduction

Myopia is a refractive error characterized by parallel rays of light focusing in front of the retina when the eyes are relaxed and light enters along the visual axis. This condition most often arises from excessive axial elongation and can also result from increased refractive power of the cornea and/or lens. When structural or positional changes in the cornea and lens predominate, the condition is termed refractive myopia and is typically evaluated using spherical equivalent refractive error (SER). In contrast, axial myopia is specifically attributable to excessive axial elongation, often quantified by the axial length (AL) of the eye (1). As excessive axial elongation elevates risks of retinal or choroidal atrophy, vitreoretinal interface traction, and various stress responses at the optic nerve head, precise assessment of axial myopia is particularly crucial.

Although AL remains the gold-standard metric for axial myopia, its interpretive value is confounded by inter-individual variability in corneal and lenticular power. Two eyes with identical ALs may differ in SER. Therefore, evaluating the severity of axial myopia still requires considering the “refractive power of the optical system.” Since the anterior corneal surface contributes the majority of the eye’s refractive power, the ratio of AL to corneal curvature radius (AL/CR) is often employed to describe the severity of axial myopia. One characteristic of axial myopia is the decrease in ocular wall stiffness caused by AL elongation. Our previous study revealed a stronger correlation between the stress-strain index (SSI) and AL/CR than between SSI and AL alone (2). However, AL/CR suffers from two notable drawbacks: 1) its unclear clinical interpretation can be opaque, and 2) its relationship with SER is non-linear (3), complicating comparisons of AL/CR among different ALs. To address these limitations, we constructed a retinal defocus distance model, ΔAL, based on the mathematical model proposed by Morgan et al., as a quantitative indicator for assessing the biomechanics of axial myopia (4–6). ΔAL represents the distance by which the eye’s focal point shifts relative to the retinal plane due to ocular elongation. However, a given ΔAL corresponds to different relative biomechanical strain in eyes of varying sizes.

Therefore, we proposed Myopic Strain, defined as the ratio of ΔAL to the eye’s focal length. By normalizing ΔAL by focal length, Myopic Strain yields a metric that compensates for eye size differences. The primary aim of this study was to develop a normalized metric that aligns with the optical and biomechanical markers of axial myopia. This investigation postulates the existence of a stable emmetropization endpoint precisely controlled by CR and ocular elongation (7), wherein the mathematical relationship among AL, CR, and SER conforms to the model established by Morgan et al. (4, 5).

## 2 Methods

### 2.1 Dataset

This cross-sectional analysis included 242 healthy young adults enrolled at the Shandong Eye Institute in Qingdao, China. The dataset included comprehensive optometric assessment after mydriasis, AL measurements obtained with the OA-2000 optical biometer (Tomey, Japan), anterior CR values (calculated as the mean of Kflat and Ksteep across a 3 mm central zone) measured by the OA-2000 (Tomey, Japan), and corneal biomechanical parameters assessed using the Corvis ST (Oculus, Wetzlar, Germany). Given the high inter-ocular parameter concordance, only left eye data were included in the analysis.

### 2.2 Derivation of Myopic Strain

Based on the mathematical model proposed by Morgan et al. (4, 5),


(1) $$\begin{array}{c} A=\frac{1}{\frac{0.22273}{k}+0.00070S+0.01368} \end{array}$$

Where A is AL (mm), k denotes the mean anterior CR (mm), and S stands for SER at the corneal plane (D).

We decomposed the total AL into an emmetropic component (AL_emmetropia_) and a defocus component (ΔAL): The theoretical AL_emmetropia_ and ΔAL are calculated as


(2) $$\begin{array}{c} AL_{emmetropia}=\frac{1}{\frac{0.22273}{CR}+0.01368} \end{array}$$

and


(3) $$\begin{array}{c} \Delta AL=AL-\frac{1}{\frac{0.22273}{CR}+0.01368} \end{array}$$

Myopic Strain was defined as the ratio of defocus distance to emmetropic length:


(4) $$\begin{array}{c} Myopic\;Stain=\frac{\Delta AL}{AL_{emmetropia}} \end{array}$$

which simplifies algebraically to


(5) $$\begin{array}{c} Myopic\;Stain=\frac{0.22273AL}{CR}+0.01368AL-1 \end{array}$$

### 2.3 Statistical analysis

**Figure 1:**
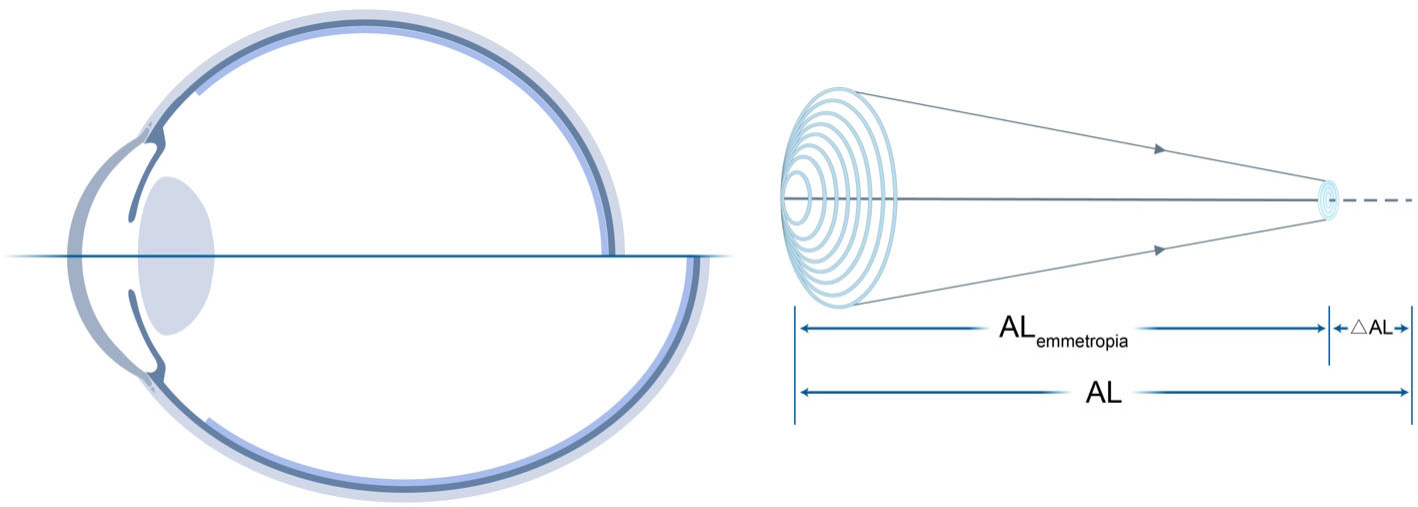
Decomposition of total AL into AL_emmetropia_ and ΔAL. Left: Schematic of AL relative to the focal plane. Right: Mathematical relationships among AL, CR, and SER, where Myopic Strain = ΔAL/AL_emmetropia_.

All analyses were conducted in R (version 4.5.0). According to the established Equations 1–3, AL was systematically divided into AL_emmetropia_ and ΔAL (**Figure 1**). A Pearson correlation analysis was initially performed to validate the relationship between SER and ΔAL. Following this confirmation, the Myopic Strain mathematical model was established in the forms of Equations 4, 5. Subsequently, additional Pearson’s correlation analyses were employed to examine the correlations among SER, SSI, Myopic Strain, and AL/CR. A comprehensive graphical matrix was constructed to analyze the intricate relationships among variables. Univariate linear regression models were utilized to estimate both unstandardized and standardized coefficients for SER as the optical feature and SSI as the biomechanical feature, using each structural metric (Myopic Strain, AL, AL/CR, and ΔAL) as predictors. Finally, comparative analysis characterized the distinctive behaviors of Myopic Strain and AL/CR relative to increasing AL values. Statistical significance was defined as *p* < 0.05.

## 3 Results

The demographic information of the subjects included in this study is displayed in **Table 1**.

**Table 1:** Characteristics of the subjects included in the study.

Characteristic	N = 242^1^
Age (year)	21.89 (7.24)
Gender	
Female	133 (55%)
Male	109 (45%)
AL (mm)	25.84 ± 1.25
CR (mm)	7.77 ± 0.30
SER (diopter)	-5.68 ± 2.34
SSI	0.84 ± 0.15

^1^Mean (SD); n (%).

AL, Axial Length; CR, Corneal Curvature Radius; SER, Spherical Equivalent Error; SSI, Stress-Strain Index.

### 3.1 ΔAL is strongly correlated with SER

**Figure 2:**
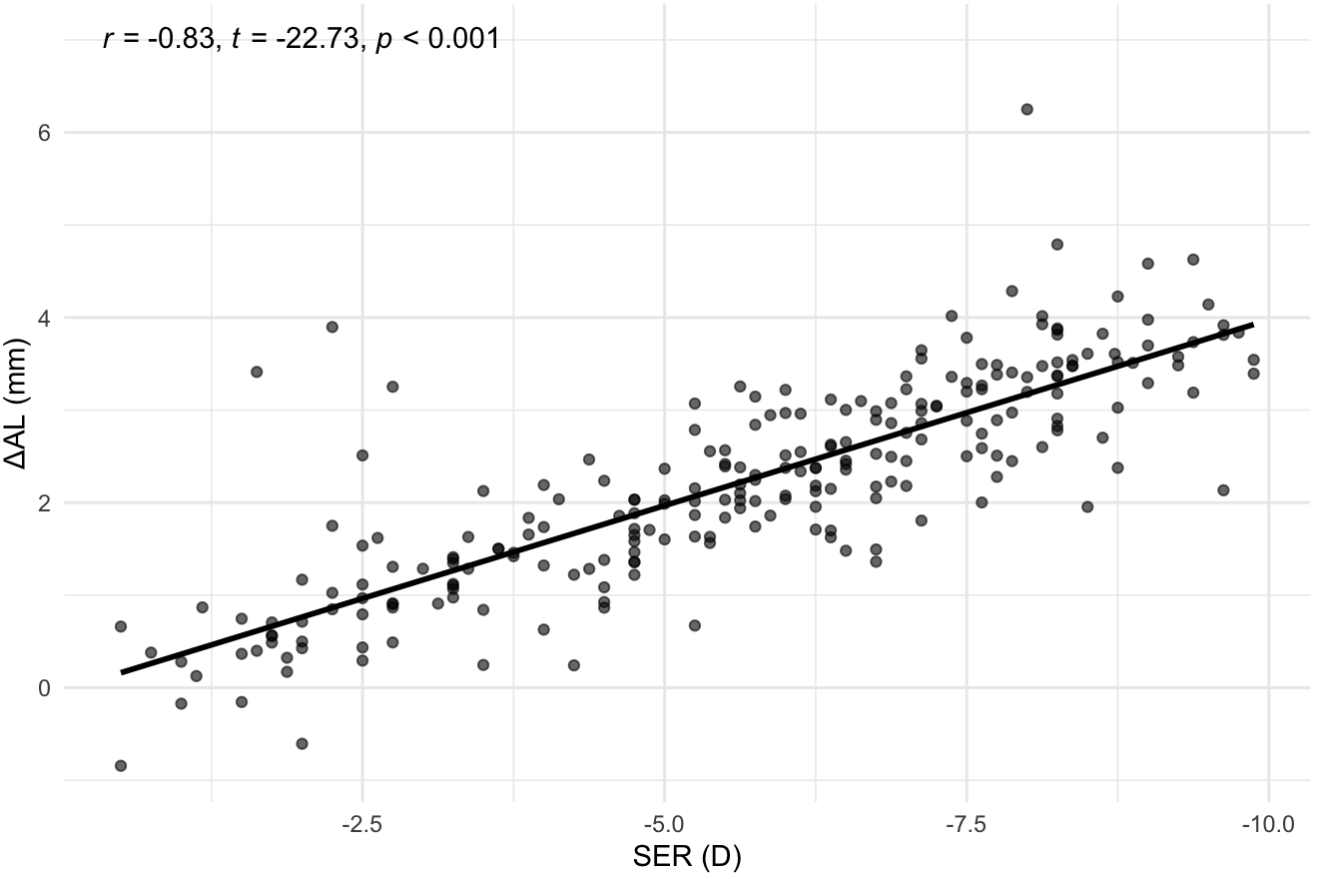
Correlation between SER and ΔAL. Scatterplot with best‐fit line. SER, Spherical Equivalent Error; ΔAL, Retinal Defocus Distance relative to Ocular Refractive Power.

To investigate the correlation between SER and ΔAL, a Pearson correlation test was conducted. The results, shown in **Figure 2**, indicated a strong negative association between SER and ΔAL (*r* = −0.83, *t* = −22.73, *p* < 0.001).

### 3.2 Myopic Strain outperforms AL/CR in explaining SER and matches its power for SSI

To compare how well Myopic Strain versus traditional metrics explain optical and biomechanical outcomes, we computed Pearson correlations and univariate regressions for each predictor (AL, AL/CR, ΔAL, and Myopic Strain) against SER and SSI.

**Figure 3:**
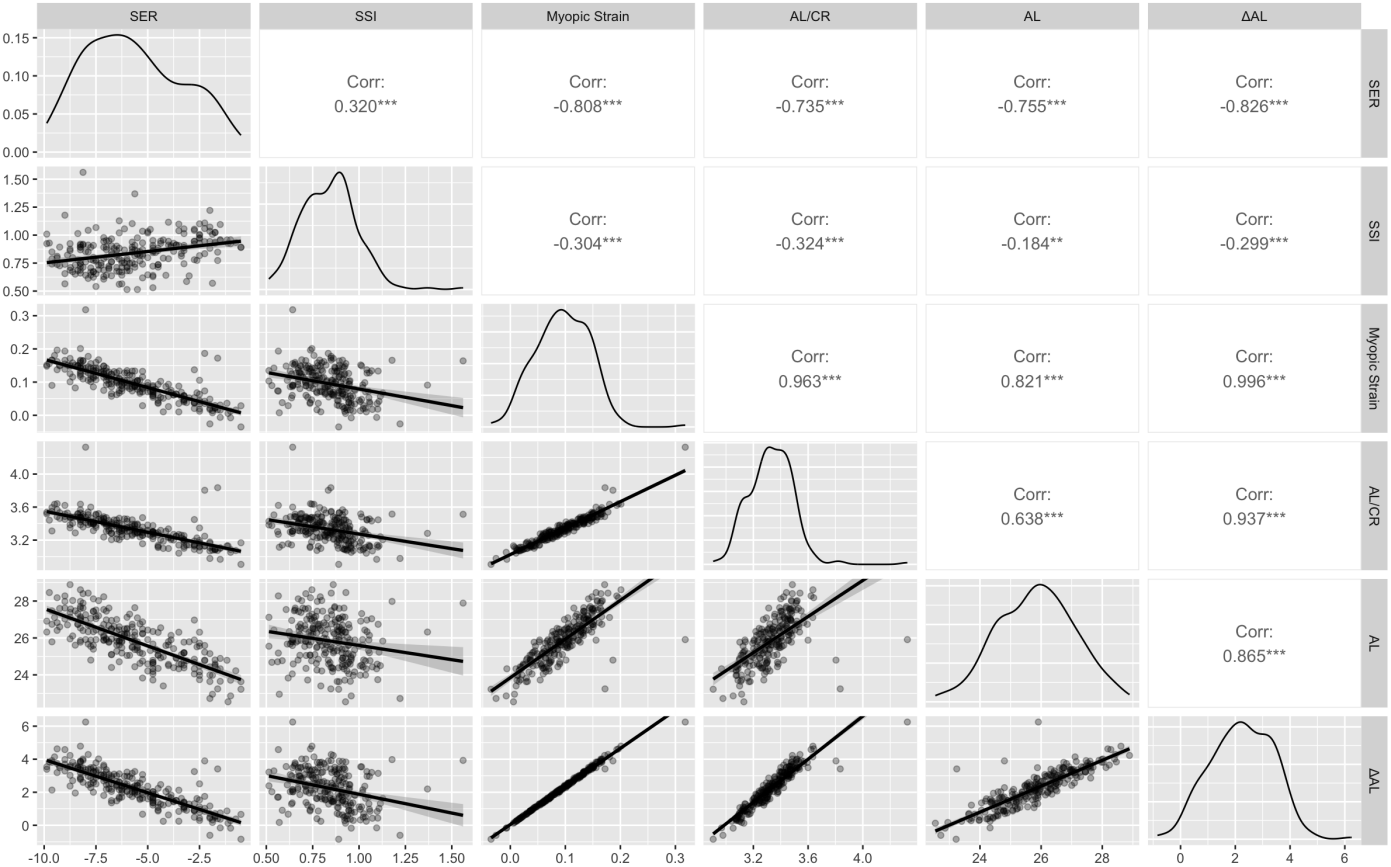
Correlation matrix for SER, SSI, Myopic Strain, AL/CR, AL, and ΔAL. Diagonal: kernel-density plots. Upper triangle: Pearson correlation coefficients (***p < 0.001). Lower triangle: scatterplots. SER, Spherical Equivalent Error; SSI, Stress-Strain Index; AL/CR, Ratio of Axial Length to Corneal Curvature Radius; AL, Axial Length; ΔAL, Retinal Defocus Distance relative to Ocular Refractive Power. **p < 0.01, ***p < 0.001.

As illustrated in **Figure 3**, the diagonal panels presented kernel-density plots, revealing the distribution characteristics and probability density functions of each variable, the upper triangular region displayed Pearson correlation coefficients with their statistical significance, while the lower triangular region illustrated scatter plots, providing an intuitive representation of the bivariate relationships between variables. The results revealed that Myopic Strain exhibited a Pearson correlation coefficient (*r*) of -0.81 (*p* < 0.001) for SER and -0.30 (*p* < 0.001) for SSI.

**Figure 4:**
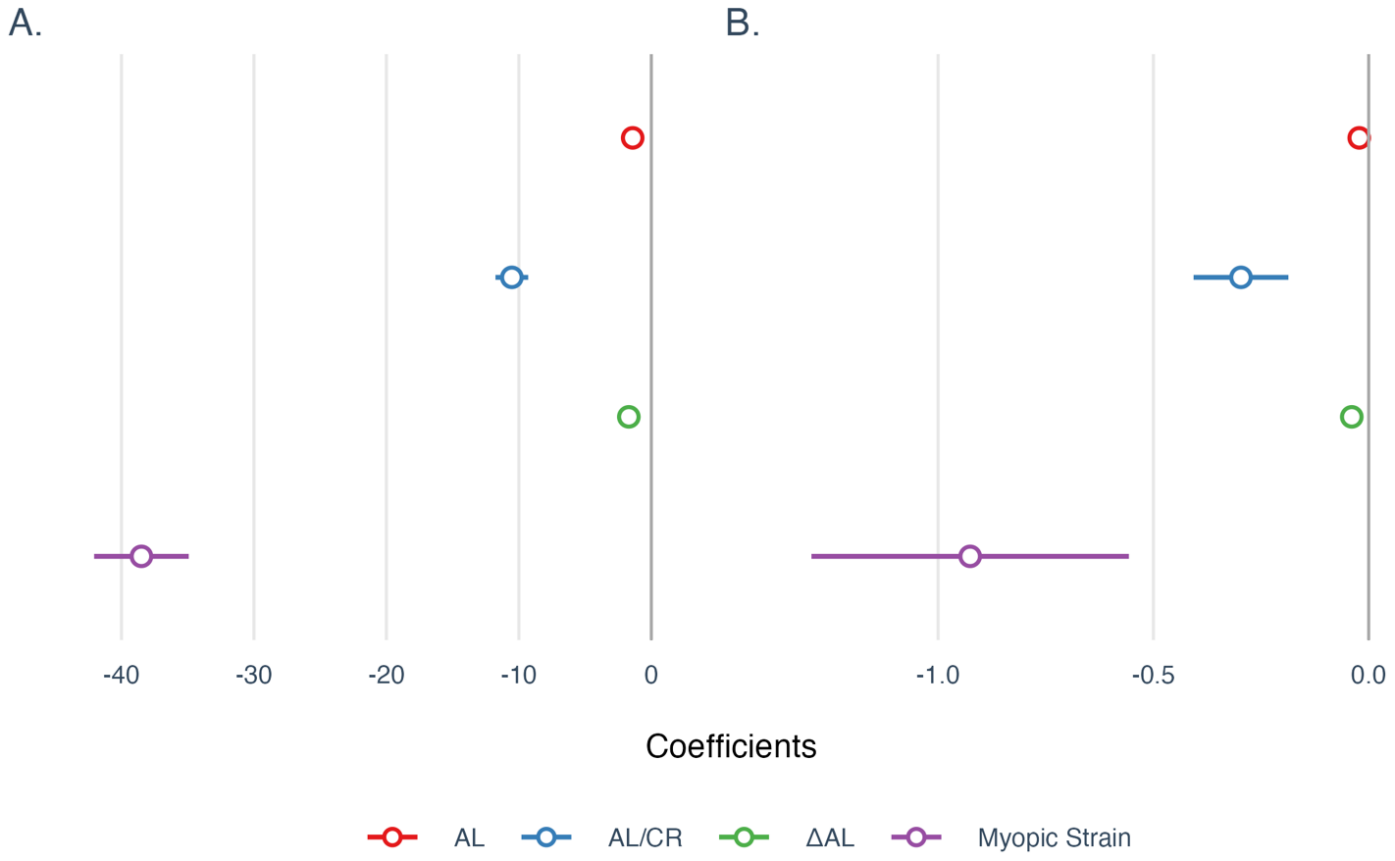
Univariate regression coefficients for models of **(A)** SER and **(B)** SSI. All coefficients were reported using unstandardized coefficients, and the 95% confidence intervals were used to describe the uncertainties of coefficients. AL, Axial Length; AL/CR, Ratio of Axial Length to Corneal Curvature Radius; ΔAL, Retinal Defocus Distance relative to Ocular Refractive Power.

Next, univariate linear regressions quantified effect sizes, reporting both unstandardized and standardized coefficients with 95% confidence intervals (CIs). In the SER model (**Figure 4A**), four different indicators (AL, AL/CR, ΔAL, and Myopic Strain) showed significant negative correlations individually. ΔAL explained the greatest variance (*R²* = 0.68, *β* = -1.70; *p* < 0.001), closely followed by Myopic Strain (*R²* = 0.65, *β* = -38.49; *p* < 0.001), both outperforming AL (*R²* = 0.57, *β* = -1.41) and AL/CR (*R²* = 0.54, *β* = -10.53) (both *p* < 0.001). The standardized coefficients highlighted ΔAL as the strongest predictor (*β* = -0.83), followed by Myopic Strain (*β* = -0.81), AL (*β* = -0.76), and AL/CR (*β* = -0.73).

In the SSI model (**Figure 4B**), the four indicators also showed negative correlations individually. AL/CR had a marginally higher explanatory power (*R²* = 0.105, *β* = -0.297; *p* < 0.001) than Myopic Strain (*R²* = 0.092, *β* = -0.926) and ΔAL (*R²* = 0.089, *β* = -0.039) (both *p* < 0.001); AL exhibited the weakest explanatory power (*R²* = 0.034, *β* = -0.022) (*p* < 0.01). While most models were statistically significant at the 0.001 level, the AL model was significant at the 0.01 level. Standardized coefficients identified AL/CR as the strongest predictor (*β* = -0.32), followed by Myopic Strain (*β* = -0.3041), ΔAL (*β* = -0.2985), and AL (*β* = -0.18).

### 3.3 Myopic Strain exhibits a strong positive linear relationship with increasing AL

**Figure 5:**
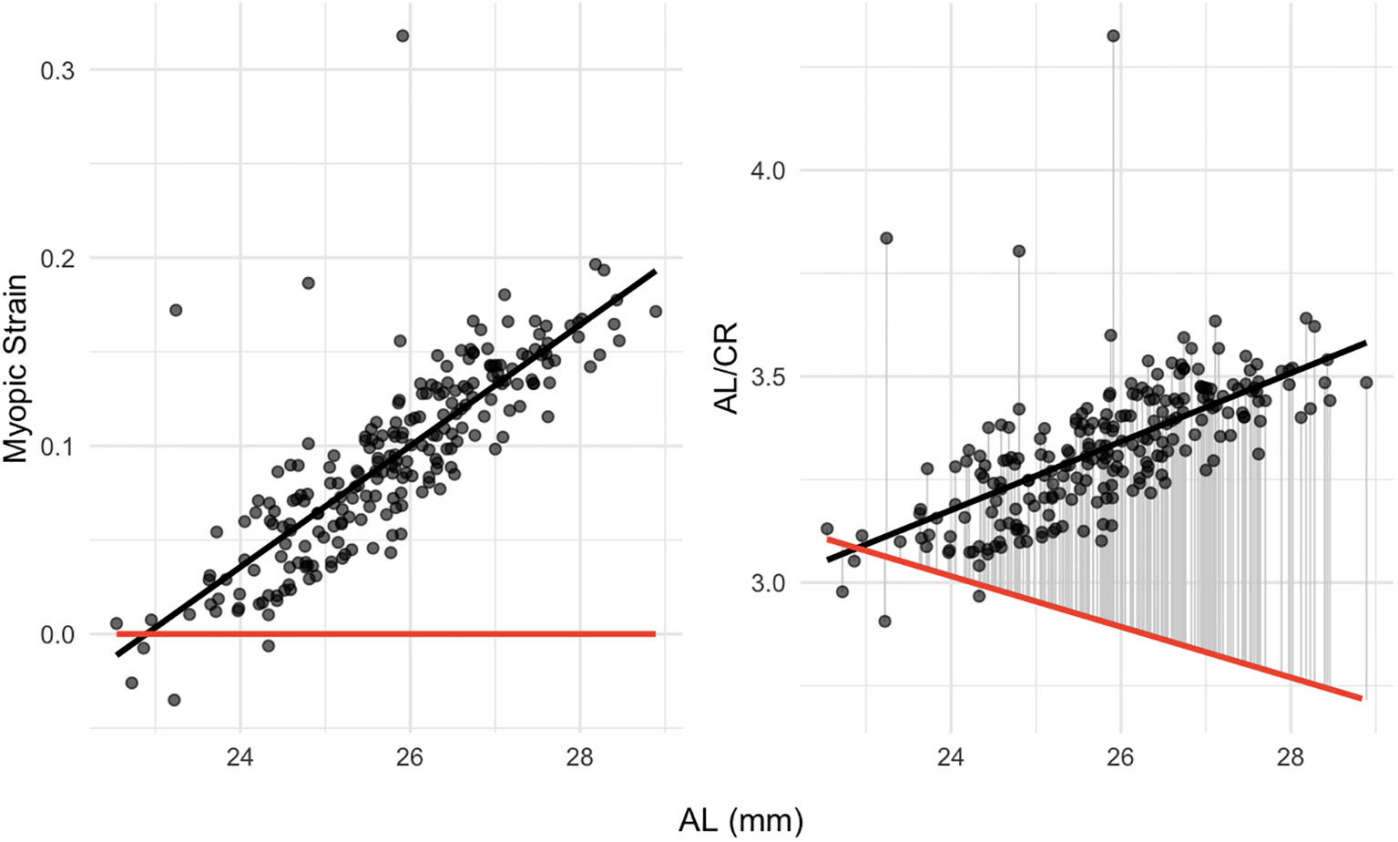
Dependence of myopic strain (left panel) and AL/CR (right panel) on AL. The red line shows the theoretical emmetropization baseline of Myopic Strain or AL/CR at SER = 0. AL, Axial Length; AL/CR, Ratio of Axial Length to Corneal Curvature Radius.

Further analysis revealed a strong positive correlation between Myopic Strain and AL (*r* = 0.82, *p* < 0.001), as well as a moderate positive correlation between AL/CR and AL (*r* = 0.64, *p* < 0.001). Under an emmetropic assumption (SER = 0), the theoretical relationship simplifies to AL/CR = (1 - 0.01368 × AL)/0.22273, yielding an inverse trend with increasing AL (**Figure 5**).

## 4 Discussion

In this study, we introduced the concept of Myopic Strain, a normalized metric that isolates the excessive component of axial elongation relative to an emmetropization baseline. Based on Morgan’s optical model (4, 5). Myopic Strain represents a normalized metric of axial elongation relative to the emmetropization baseline, serving as an appropriate metric for comprehensively evaluating axial myopia progression.

SER reflects the optical severity of myopia, and SSI assesses the biomechanical stiffness of the ocular wall, which diminishes during axial elongation. These two dimensions capture complementary dimensions of axial myopia. In head-to-head comparisons, Myopic Strain demonstrated the strongest explanatory power for SER, outperforming both AL and AL/CR, and nearly matched AL/CR for SSI.

Given that CR accounts for the majority of the eye’s refractive power, AL/CR approximates the ratio of AL to ocular focal distance. However, for myopic eyes, AL comprises two components: the focal distance adaptive to refractive power (AL_emmetropia_) and the defocus distance (ΔAL). By distinctively separating these components, Myopic Strain mathematically corrects the AL/CR bias across different eye sizes (intuitively demonstrated in algebraic form in formula (5)). Theoretically, as axial myopia progresses, the increasing proportion of ΔAL renders Myopic Strain increasingly appropriate for evaluating axial myopia. Moreover, Myopic Strain offers more intuitive interpretability compared to AL/CR in practical contexts. Nevertheless, it is important to acknowledge that AL/CR remains computationally simpler and more readily applicable in real-world scenarios.

Specific genetic loci have been shown to co‐regulate CR and eye elongation to maintain emmetropic status (7). However, during the myopic progression, ocular refractive power may dynamically adapt to compensate for axial elongation. These adaptive responses could lead our calculated focal distance to incorporate some degree of axial elongation, thereby underestimating the reduction in scleral biomechanical strength associated with axial myopia. Therefore, future research should combine Myopic Strain with genetically informed models of ocular development to more comprehensively assess axial myopia progression.

In conclusion, Myopic Strain provides a size-normalized framework for quantifying axial myopia progression. It outperforms traditional metrics in explaining refractive error and matches them in capturing biomechanical changes, thereby offering a tool for research into myopia pathophysiology.

## Data Availability

The raw data supporting the conclusions of this article will be made available by the authors, without undue reservation.
